# Institutional Laundries

**Published:** 1912-04-27

**Authors:** 


### INSTITUTIONAL LAUNDRIES.

In a recent number of the *Zeitschrift für Krankenanstalten*, Herr Klaffke, the superintendent of the Elberfeld Municipal Hospital, writes at some length on the subject of the institutional laundry, approaching the matter from a purely practical point of view. His article is almost exhaustive and teems with informative hints and suggestions. First he deals with the general question of loss and deterioration. We all know how much in private life we suffer from the washerwoman—now more often the laundry machine. The tearing up of cotton and linen goods, the unravelling of mixed material, and the general damaging of stiff goods are everyday commonplace grievances which are, however, far more serious in institutional than in private life. That they occur is due commonly to carelessness and want of adequate supervision. In an institutional laundry, properly equipped and staffed, such carelessness is inexcusable. For, as Herr Klaffke shows, nearly every variety of destruction can be prevented by proper care and management. One of the most common causes of indifferent laundry work in English institutions is the use of hard or insufficiently soft water for washing purposes; another equally material cause is false economy in the use of soap. By sparing the soap the material washed almost invariably runs the risk of being spoiled in a greater or lesser degree. We are too prone to look upon soap merely as a chemical means of removing dirt, whereas in these days, when comparatively heavy-working rinsing and mangling machinery are used, a good soap is invaluable as a mechanical lubricant in preventing damage to the linen by meliorating the action of the rollers. This lubricating action can, however, only be fully secured when soft water is employed; with hard waters a large amount of soap is wasted. Hospital workers in general have no conception of what this waste really means, nor is it easy to express it in figures. But, as a general rule, the experiments at the large Rudolf Virchow Hospital have shown that one part of lime in 100,000 parts of water means sixteen times the amount of soap, so far as laundry purposes is concerned, that is used up in soft water containing no lime. A certain percentage of this loss can be spared by a liberal use of washing soda, but by far the best means of securing economy of soap is by previously softening the water, and undoubtedly the most practical method of softening is the so-called Permutite process. A very interesting point to which Herr Klaffke draws attention is the damaging action upon cotton and linen goods of electric bleaching. Recent experiments have shown conclusively that these methods of whitening tend to deteriorate the quality of the articles subjected to them by as much as 50 per cent. For this reason, when the method is used, care must be taken to employ very weak solutions, to turn the articles frequently so as to ensure an even action, and to exercise discrimination in selecting articles for bleaching by this method. The damage done by the use of strong chemical adjuvants, now so frequently used where quick washing is required, is also very great. Soaps containing a relatively low percentage of fat and alkaline soap-powders appear to be responsible for most of the damage done in institutional laundries. Where chemical disinfectants are used the damage is much greater, although not in general so apparent at first. Rapid steam boiling, for example, tends to deteriorate the fibre of cotton articles to a small extent; if to the wash-water a percentage of an alkaline soap-powder is added, this deteriorating effect is markedly increased, whereas if a superfatted soap is used the destructive action is far less.

We do not know of any similar series of experiments to these in English institutions. It would be instructive and interesting, therefore, to have the views of hospital workers who have experience of institutional laundry work on the subject for purposes of comparison. More especially is it desirable to find out what are the most economical and efficient methods of softening hard water employed in our large institutions. We shall be grateful to readers who can supply us with the information with a view to publication in the form of an extended comparison of results.

